# Predictive Factors for Urinary Tract Infections in Patients with Type 2 Diabetes

**DOI:** 10.3390/jcm13247628

**Published:** 2024-12-14

**Authors:** Teodora Sorescu, Andrei Cosnita, Adina Braha, Romulus Timar, Bogdan Timar, Monica Licker, Sandra Lazar, Laura Gaita, Oana Albai, Simona Popescu

**Affiliations:** 1 Second Department of Internal Medicine: Diabetes, Nutrition, Metabolic Diseases, and Systemic Rheumatology, “Victor Babes” University of Medicine and Pharmacy, 300041 Timisoara, Romania; sorescu.teodora@umft.ro (T.S.); braha.adina@umft.ro (A.B.); timar.romulus@umft.ro (R.T.); bogdan.timar@umft.ro (B.T.); gaita.laura@umft.ro (L.G.); albai.oana@umft.ro (O.A.); popescu.simona@umft.ro (S.P.); 2 Department of Diabetes, Nutrition and Metabolic Diseases, “Pius Brînzeu” Emergency Clinical County Hospital, 300723 Timisoara, Romania; 3 Centre for Molecular Research in Nephrology and Vascular Disease, “Victor Babes” University of Medicine and Pharmacy, 300041 Timisoara, Romania; sandra.lazar@umft.ro; 4 Department IX, Surg & Ophthalmol, “Victor Babes” University of Medicine and Pharmacy, 300041 Timisoara, Romania; 5 Microbiology Department, Multidisciplinary Research Center of Antimicrobial Resistance, “Victor Babes” University of Medicine and Pharmacy, 300041 Timisoara, Romania; licker.monica@umft.ro; 6 Microbiology Laboratory, “Pius Brinzeu” Emergency Clinical County Hospital, 300723 Timisoara, Romania; 7 First Department of Internal Medicine, “Victor Babes” University of Medicine and Pharmacy, 300041 Timisoara, Romania; 8 Doctoral School of Medicine, “Victor Babes” University of Medicine and Pharmacy, 300041 Timisoara, Romania; 9 Department of Hematology, Emergency Municipal Hospital, 300254 Timisoara, Romania

**Keywords:** urinary tract infections, type 2 diabetes, risk factors, predictive factors, SGLT-2 inhibitors

## Abstract

**Background/Objectives:** Patients with diabetes (DM) are at an increased risk of infection, with urinary tract infections (UTIs) being common among individuals with type 2 DM (T2D). The aim of this study was to determine the prevalence and risk factors for UTIs among hospitalized T2D patients from Timișoara, Romania. **Methods:** The hospital records of 1139 T2D adult inpatients who were ordered to provide urine cultures during hospitalization were reviewed. **Results:** The prevalence of UTIs among T2D patients was 19.7%, and was higher in women than in men (27.5% vs. 9.8%, *p* < 0.0001). Patients with UTIs presented a significantly older age, a longer duration of DM, a higher BMI, higher levels of HbA1c, higher renal function parameters, and more frequent DM-related complications and comorbidities than patients without UTIs. The following predictors were associated with increased UTI risk: age (OR = 1.05, *p* < 0.0001); duration of DM (OR = 1.04, *p* < 0.0001); BMI (OR = 1.05, *p* < 0.0002); HbA1c levels (OR = 1.58, *p* < 0.0001); female gender (OR = 3.47, *p* < 0.0001); and the presence of retinopathy (OR = 1.47, *p* = 0.0118), chronic kidney disease (OR = 3.98, *p* < 0.0001), distal symmetric polyneuropathy (OR = 7.65, *p* < 0.0001), and cerebrovascular disease (OR = 4.88, *p* < 0.0001). The use of sodium-glucose co-transporter 2 (SGLT2) inhibitors did not influence the risk of developing UTIs. **Conclusions:** T2D patients with prolonged disease duration, poor glycemic control, and DM-related complications are at an increased risk of developing UTIs. Therefore, a targeted therapeutic strategy addressing these risk factors is essential.

## 1. Introduction 

Type 2 diabetes (T2D) is a chronic metabolic disease characterized by hyperglycemia due to insulin resistance and progressive beta-cell dysfunction, accounting for over 90% of diabetes mellitus (DM) cases worldwide [1]. The global prevalence of T2D has been rising, driven by factors such as urbanization, an aging population, and unhealthy lifestyle patterns [2]. 

Long-standing and poorly controlled DM can determine serious life-threatening complications, such as cardiovascular disease, kidney failure, and diabetic retinopathy, leading to blindness, peripheral neuropathy, and lower-limb amputations [3], ultimately reducing the quality of life and driving up healthcare costs [4]. 

Patients with DM are at increased infection risk [5] due to multiple factors, including impaired immune function and chronic hyperglycemia, which affects neutrophil activity and reduces the immune system’s efficiency [6,7]. Urinary tract infections (UTIs) are a common complication among individuals with T2D [8]. Several factors contribute to an elevated risk of uropathogenic infections in diabetic patients, and among them, hyperglycemia and altered immune response are the most important [9]. Hyperglycemia leads to glycosuria, which represents a favorable environment for the colonization of urine with microorganisms. Also, the hyperglycemic environment is responsible for immune function impairment in diabetic patients, alteration of polymorphonuclear leukocyte function and adhesion, chemotaxis and phagocytosis, and lower levels of urinary interleukin concentrations (IL-6 and IL-8) [6,10,11]. Moreover, UTIs are not only more frequent but often more severe in diabetic patients, leading to complications such as emphysematous cystitis and pyelonephritis, renal abscesses and renal papillary necrosis, bacteremia, and increased mortality [9,10].

Several factors were found to increase the risk of UTIs for diabetic patients, including advanced age, poor metabolic control, and long-term complications, especially diabetic nephropathy and cystopathy [9,10]. Moreover, concerns have recently been raised regarding the infection-related safety profile of sodium-glucose co-transporter 2 (SGLT2) inhibitors, which are a relatively novel class of glucose-lowering agents for treating T2D. SGLT2 inhibitors suppress renal glucose reabsorption, leading to increased urinary glucose excretion and ameliorating hyperglycemia [12]. Also, SGLT2 inhibitors have demonstrated significant benefits in slowing the progression of CKD and in lowering the risk of kidney failure and all-cause mortality, cardiovascular death, and hospitalization for heart failure [11]. More recently, there has been evidence regarding the potential neuroprotective effects of SGLT2 inhibitors in adult patients [13]. There are studies that have found an association between the use of SGLT2 inhibitors and genital tract infections [12,14,15,16], with some reports also linking their use to an increased risk of UTIs [12,14]. This effect may be attributed to glucosuria, a consequence of the treatment with these glucose-lowering agents [12].

Given the significant healthcare burden associated with UTIs in the diabetic population, understanding the factors that predispose these individuals to infections is critical for developing targeted prevention and management strategies. Although there are many studies in the literature exploring the risk factors for UTIs in patients with DM, only a few have been conducted in our country [17]. Therefore, we have conducted the present research to evaluate the prevalence of UTIs among hospitalized patients with T2D from Timișoara, Romania, and to identify the main factors associated with the occurrence of UTIs in this category of patients.

## 2. Materials and Methods

### 2.1. Study Design, Area, and Population

We conducted an observational, retrospective study in the Diabetes, Nutrition, and Metabolic Diseases (DNMD) Clinic from the ‘Pius Brînzeu’ Emergency Clinical County Hospital Timișoara, Romania, between January 2023 and December 2023. This institution is a tertiary-care university-affiliated teaching hospital with a capacity of 1174 beds, providing healthcare services for the western region of Romania. 

The study inclusion criteria were T2D patients aged 18 years and older, who were hospitalized in the DNMD Clinic and had urine cultures collected and analyzed during hospitalization. Patients with a type of DM other than T2D, those who had used antibiotic therapy in the last 14 days before the admission, and those who had not been ordered to provide urine cultures during hospitalization were excluded. 

The Hospital Ethical Committee approved the study (approval no. 487/25 September 2024). 

### 2.2. Data Collection

Data were collected from patients’ hospital records. They included demographics (gender and age), anthropometric characteristics (height, weight, and body mass index—BMI), and data about T2D. Regarding T2D, we evaluated the duration of the disease, treatment, and glycated hemoglobin—HbA1c—to establishing the glycemic control. The presence of chronic complications of DM (chronic kidney disease—CKD; retinopathy; cerebrovascular disease; coronary artery disease; peripheral artery disease; and distal symmetric polyneuropathy—DSPN) and comorbidities commonly associated with T2D (hypertension, dyslipidemia, and overweight and obesity) were also assessed. Overweight and obesity were defined according to World Health Organization (WHO) criteria as a BMI between 25 and 29.9 kg/m^2^ for overweight and >30 kg/m^2^ for obesity, respectively [18]. Data regarding the renal function (serum creatinine; blood urea nitrogen—BUN; uric acid; estimated glomerular filtration rate—eGFR; using the CKD-EPI creatinine equation 2021; and the urine albumin–creatinine ratio—uACR), the lipid profile (total cholesterol; high-density lipoprotein cholesterol—HDLc; triglycerides—TGs; and low-density lipoprotein cholesterol—LDLc), and information related to UTI diagnosis were also acquired. The treating physician determined the diagnosis of UTIs and the need for a urine culture during hospitalization. In the hospital’s Microbiology Laboratory, the cut-off value for a positive urine culture is the presence of more than 10^5^ colony-forming units/mL (CFU/mL) of urine.

### 2.3. Statistical Analysis

Analysis was performed using MedCalc^®^ Statistical Software version 23.0.5 (MedCalc Software Ltd., Ostend, Belgium; https://www.medcalc.org; 2024). Data are reported as medians with interquartile ranges or average ranks for continuous variables and as frequencies with percentages for categorical variables. The Mann–Whitney U test was used to assess differences between groups for continuous variables, while the Chi-square test analyzed categorical variables. The odds ratio (OR) was calculated to estimate the risk of UTIs occurring following exposure or non-exposure to a specific factor. For cases where exposure was quantified with continuous variables, a logistic regression model was applied to examine risk factors. Predictor selection for the model was conducted through a stepwise consecutive–prospective acceptance approach, including predictors with a significance level of *p* ≤ 0.1. Receiver-operating characteristic (ROC) curve analysis was performed to evaluate the predictive ability of specific factors for UTIs. This method was used to assess the predictors’ sensitivity, specificity, and overall diagnostic performance. To quantify the overall accuracy of specific factors as predictive markers for UTIs, we calculated the area under the ROC curve (AUC), with an AUC of 1 indicating perfect discrimination and an AUC of 0.5 suggesting no discriminative ability. We then identified the optimal threshold of the predictors using the Youden index. The present study’s statistical significance threshold was set at a *p*-value of less than 0.05.

## 3. Results

### 3.1. Characteristics of the Study Group

Of the 1374 patients with T2D admitted to the DNMB Clinic from Timisoara between January and December 2023, 1139 (82.8%) had urine cultures collected and analyzed and were enrolled in the study. Among them, 225 (19.7%) patients had positive samples for uropathogens, indicating a prevalence of UTIs of 19.7% (95% CI: 17.2–22.5%), which was higher in women than in men (27.5% vs. 9.8%, *p* < 0.0001).

The characteristics of the T2D patients from the study group are presented in Table 1.

### 3.2. Factors Associated with the Presence of UTIs in T2D Patients

When comparing T2D patients with and without UTIs, we found that patients with UTIs had a significantly older age (70 years vs. 62 years, *p* < 0.0001), a longer duration of DM (13 years vs. 9 years, *p* < 0.0001), a higher BMI (31 kg/m^2^ vs. 30 kg/m^2^, *p* = 0.01), higher levels of HbA1c (9% vs. 8%, *p* < 0.0001), and lower HDLc levels (40 mg/dL vs. 42 mg/dL, *p* = 0.03) than patients without UTIs. Also, the renal function parameters (serum creatinine, BUN, uric acid, and uACR) were significantly higher in patients with UTIs than in those without (Table 2).

The analysis of DM-related complications and comorbidities revealed statistically significant differences in relation to the presence or absence of UTIs. Thus, diabetic retinopathy (39.6% vs. 30.7%, *p* = 0.01), CKD (40.4% vs. 14.6%, *p* <0.0001), DSPN (94.2% vs. 68.1%, *p* < 0.0001), cerebrovascular disease (23.1% vs. 5.8%, *p* < 0.0001), and hypertension (92.9% vs. 84.1%, *p* = 0.0007), respectively, were more frequently observed in patients with UTIs compared to those not presenting with a UTI (Table 3).

Furthermore, we wanted to evaluate whether the treatment of T2D with an SGLT2 inhibitor is associated with the presence of UTIs. Of the total number of T2D patients included in the study group, 21.8% (248/1139) were treated with SGLT2 inhibitors. Among patients who presented with a UTI, 25.3% (57/225) were treated with SGLT2 inhibitors, while among patients without UTIs, 20.9% (191/914) were using SGLT2 inhibitors for the treatment of T2D (OR 1.28; 95% CI: 0.91–1.80, *p* = 0.1).

### 3.3. Risk Factors for UTIs in Patients with T2D

The logistic regression model, which was employed to analyze the relationship between various clinical parameters and the occurrence of UTIs, identified age (OR = 1.05, *p* < 0.0001), duration of DM (OR = 1.04, *p* < 0.0001), BMI (OR = 1.05, *p* < 0.0002), and HbA1c levels (OR = 1.58, *p* < 0.0001) as significant risk factors for UTIs. Moreover, female gender (OR = 3.47, *p* < 0.0001) and the presence of retinopathy (OR = 1.47, *p* = 0.0118), CKD (OR = 3.98, *p* < 0.0001), DSPN (OR = 7.65, *p* < 0.0001), and cerebrovascular disease (OR = 4.88, *p* < 0.0001), respectively, were other significant factors associated with the risk of occurrence of UTIs. The treatment of T2D with an SGLT2 inhibitor did not influence the risk of UTIs in our analysis (Table 4).

**Figure 1:**
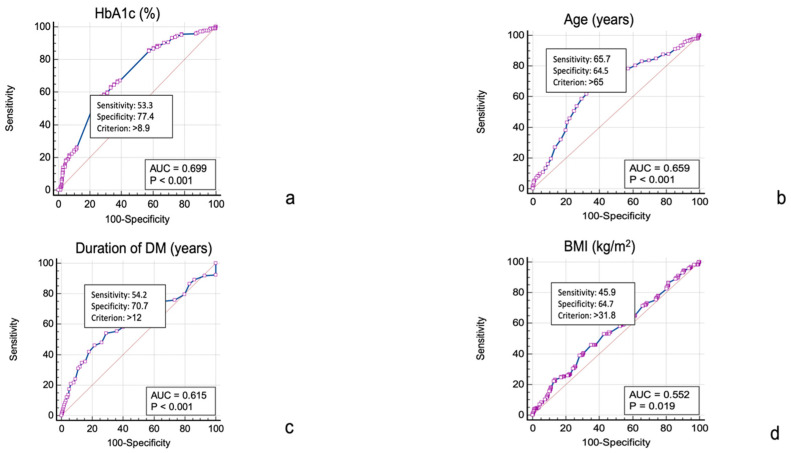
ROC curve analysis for evaluating the performance of (**a**) HbA1c (AUC = 0.699), (**b**) age (AUC = 0.659), (**c**) duration of DM (AUC = 0.615), and (**d**) BMI (AUC = 0.552) in predicting UTIs in patients with T2D.

We conducted an ROC curve analysis to assess the diagnostic performance of several clinical parameters in identifying patients with UTIs. Among the evaluated parameters, HbA1c levels demonstrated the highest discriminative ability, showing a sensitivity of 53.3% and a specificity of 77.4% (AUC = 0.699, *p* <0.001) at a threshold of >8.9%. Age and duration of DM were other valid predictors for UTIs in T2D patients, while BMI values showed borderline discriminative ability in predicting UTIs (Figure 1). According to the Youden index for the age variable, a patient’s age being higher than 65 years was a valid predictor for the occurrence of UTIs; this threshold had a sensitivity of 65.7% and a specificity of 64.5%, with an AUC of 0.659 (*p* < 0.001). The duration of DM proved to be a valid predictor for the occurrence of UTIs. According to the model built with DM as a predictor for the development of UTIs, the threshold with the best performance was a duration of DM longer than 12 years; this threshold had a sensitivity of 54.2%, a specificity of 70.7%, and a corresponding AUC of 0.615 (*p* < 0.001). BMI was also marginally associated with an increased risk of UTI occurrence, with an AUC of 0.552 (*p* = 0.019), a BMI threshold of 31.8 kg/m^2^, a sensitivity of 45.9%, and a specificity of 64.7%.

## 4. Discussion

This study aimed to identify the key risk factors for UTI occurrence in patients with T2D. Among all participants, 19.7% had a UTI, which is a finding consistent with existing data from the literature. Thus, a significant meta-analysis performed by Salari et al. [11], which analyzed 15 studies with 827,948 participants with T2D, found a UTI prevalence rate of 11.5%. In a comparable study from Portugal, which included 7347 patients, a similar prevalence of UTIs among individuals with T2D was observed [19].

In our study, we observed a statistically significantly higher prevalence of UTIs in women compared to men. The pathogenic mechanisms behind this difference can be attributed to various anatomical factors. Women are generally at a higher risk of UTIs due to their shorter urethra, which facilitates easier bacterial access to the bladder, increasing the likelihood of infections after bowel movements or intercourse [9]. However, some studies suggest that men may face a greater UTI risk potentially due to prostate volume, which is linked to benign prostatic hyperplasia—a known risk factor for UTIs [20]. In the study by Carrondo et al. [19], the UTI rate was significantly higher in women (23.6%) than in men (10.5%), a finding consistent with our study. Female gender was found to increase the risk for UTIs in other previous reports [21,22]. 

When comparing T2D patients with and without UTIs, we found a significantly higher BMI in UTI patients. The link between BMI and the risk of UTIs has been explored in various reports. A significant meta-analysis encompassing 19 studies [23] identified a clear association between BMI and UTI incidence. The analysis revealed that individuals with obesity had a significantly higher risk of developing UTIs compared to those without obesity (RR = 1.45; 95% CI: 1.28–1.63; *p* < 0.001). The conclusion that obesity significantly raises the risk of UTIs is not surprising, as it aligns with the findings of other studies on the topic [24,25]. The exact mechanisms linking obesity to an increased risk of UTIs remain unclear. One possible explanation involves impaired immune responses in obese individuals. Adipose tissue secretes various adipokines, such as leptin, adiponectin, and pro-inflammatory cytokines, like TNF-α, IL-6, and IL-1β, all of which play key roles in immune regulation [26,27]. In obesity, leptin resistance, driven by hyperleptinemia, may weaken immune function, while low adiponectin levels can impair the activity of killer cells and cytokine production. Additionally, the elevated levels of pro-inflammatory cytokines associated with obesity can trigger systemic inflammation, which may reduce the body’s ability to resist infections [23,28].

Age was another predictor for UTIs in our study, with T2D patients with UTIs being significantly older than those without UTIs (70 years vs. 62 years, *p* < 0.0001). The association between age and increased UTI risk aligns with existing findings in the literature [8,19,22].

Moreover, T2D patients included in the present analysis had a median disease duration of 9 years and poor glycemic control, as reflected by a median HbA1c value of 8%. Both factors were strong predictors of the occurrence of UTIs. It is known that glycemic control is closely related to UTI appearance. Elevated glucose levels in the urine may promote the growth of pathogenic bacteria [9,29,30]. Factors related to the patient, including age, metabolic control, and the duration of DM, have been identified as potential contributors to an increased risk of infection in individuals with DM [9,29]. A weakened immune response may also reduce the patient’s ability to combat bacterial growth [6,29]. Elevated glucose levels in the renal parenchyma provide an ideal environment for microbial growth and multiplication, potentially contributing to the development of pyelonephritis and complications like emphysematous pyelonephritis [31]. Therefore, obtaining and maintaining the therapeutic targets for optimal glycemic control is mandatory. Moreover, drinking adequate amounts of fluids to ensure good urine flow, and respecting the hygiene measures are important therapeutic strategies to prevent UTIs.

In our study group, patients with UTIs had a longer DM duration than those without UTIs. A prolonged duration of DM is known to increase the risk of developing chronic complications. Notably, retinopathy, CKD, DSPN, and cerebrovascular disease were identified as significant factors associated with the occurrence of UTIs in our study. 

Several reports have highlighted the relationship between CKD and UTIs [32,33]. In a study by Wang et al. [32], it was found that the prevalence of UTIs in CKD patients was three times higher compared to those without CKD. Patients with UTIs are often treated with nephrotoxic antibiotics, which can contribute to kidney damage [34]. Moreover, individuals with CKD are at a higher risk of developing UTIs, potentially due to the immunodeficiency commonly seen in CKD patients [33]. In these patients, the immune system is altered; the most important aspects are represented by elevated levels of TNFα and IL-6, as well as the increased apoptosis of T lymphocytes CD4+ [35]. A study conducted by Ishigami et al. [36] showed an association between high levels of TNFα, IL-6, and C-reactive protein, as well as the risk of hospitalization for severe infections, including UTIs. Genetic factors are also involved in the occurrence of UTIs in patients with CKD, a fact that is highlighted by the familial aggregation of this infection [35]. In addition, proteinuria, which is very frequent in CKD patients, is a risk factor for infectious diseases [35].

The relationship that we found between cerebrovascular disease and the risk of UTIs was also demonstrated by other authors [19]. Several factors identified in patients with cerebrovascular disease may explain this association, such as the high incidence of bladder dysfunction following an acute ischemic stroke, the frequent urinary catheterization encountered in this category of patients, and also the poor hygiene associated with post-stroke disability [37,38]. 

DSPN was also identified as a significant risk factor for UTIs in our study. DSPN, the most common form of neuropathy in patients with DM, was present in 94.2% of patients with T2D and UTIs from our study. DSPN may be associated with autonomic neuropathy, which may cause genitourinary disturbances, including sexual dysfunction and bladder dysfunction [39]. Diabetic cystopathy can lead to incomplete bladder emptying, urinary retention, and a higher residual urine volume, creating an environment conducive to bacterial growth and increasing the risk for recurrent UTIs [40]. A recent meta-analysis, including more than 4000 patients, identified the presence of neuropathy as a risk factor for asymptomatic bacteriuria in patients with T2D [41].

Our study also identified diabetic retinopathy as a risk factor for UTIs, a result which is consistent with other previous findings [42,43]. 

The association of SGLT2 inhibitors with the risk of UTIs is controversial and requires additional investigations. A possible explanation for different results in the literature may be the fact that there are several potential factors involved in UTI occurrence in patients treated with SGLT2 inhibitors, like the agent used and the dosage administered [14]. In our study group, the use of SGLT2 inhibitors for treating T2D was not associated with the presence of UTIs, nor did it influence the risk of developing UTIs. This finding aligns with studies that indicate that while SGLT2 inhibitors are linked to a higher risk of genital infections, they do not significantly elevate the risk for UTIs [15,16]. 

The present study boasts several strengths, including a large, representative sample size that effectively reflects the health status of diabetic patients with UTIs in our region. Nonetheless, certain limitations should be acknowledged. The retrospective nature of the study and the single-center design may restrict our findings’ generalizability and applicability to broader therapeutic recommendations. While we employed multivariate logistic regression, some factors, such as genetic predispositions and environmental influences, were not included. To address these gaps, future research should adopt longitudinal, multi-center approaches. At the same time, considering the study design and large sample, the causality relationship between the significant associations may not always be suggested, considering that in some aspects the study may be overpowered and thus may lack confidence. However, this study’s main aim is not to demonstrate causality relationships but rather to raise awareness among healthcare professionals that UTI development in diabetic patients is influenced both by conventional factors, such as glycemic control and the duration of DM, as well as broader systemic pathologies. Understanding these influences is crucial for optimizing patient care and enhancing quality of life.

## 5. Conclusions

Our findings align with the existing literature, highlighting that female gender, age, poor glycemic control, and prolonged DM duration are among the key risk factors for UTIs in T2D patients. This study also identified retinopathy, CKD, DSPN, and cerebrovascular disease as the most significant comorbidities associated with UTI occurrence and progression. Optimal glycemic control remains fundamental in preventing UTIs, with lifestyle changes including diet and physical activity being the main steps for managing DM complications and comorbidities. Based on our findings, a risk stratification of these patients could be achieved in order to identify those at a higher risk of infection. We suggest that for DM patients who have the identified risk factors, a routine urine analysis or urine culture should be performed. All these recommendations are crucial for reducing UTI incidence and improving patient outcomes.

## Figures and Tables

**Table 1:** Characteristics of the studied patients.

Variable	Overall	Women	Men	*p*-Value *
n (%)	1139	641 (56.3%)	498 (43.7%)	
Age ^a^ (years)	63.0 (57.0; 72.0)	65.0 (617.2)	61.0 (509.1)	<0.0001
Diabetes duration ^a^ (years)	9.0 (4.0; 15.0)	10.0 (606.8)	8.0 (522.5)	<0.0001
BMI ^a^ (kg/m^2^)	30.0 (26.5; 34.0)	30.8 (612.2)	29.0 (514.5)	<0.0001
HbA1c ^a^ (%)	8.0 (7.4; 9.0)	8.0 (607.3)	8.0 (521.9)	<0.0001
LDL ^a^ (mg/dL)	108.0 (83.2; 138.0)	109.0 (592.2)	106.0 (541.3)	0.009
HDL ^a^ (mg/dL)	42.0 (35.0; 49.0)	43.0 (599.0)	41.0 (531.5)	0.0006
TGs ^a^ (mg/dL)	168.0 (118.0; 242.0)	172.0 (583.1)	159.0 (553.0)	0.1
BUN ^a^ (mg/dL)	40.0 (31.0; 53.0)	41.0 (576.9)	40.0 (561.0)	0.4
Serum creatinine ^a^ (mg/dL)	0.9 (0.8; 1.1)	0.9 (523.3)	1.0 (630.0)	<0.0001
eGFR ^a^ (mL/min/1.73 m^2^)	75.0 (59.5; 95.0)	72.0 (517.5)	81.0 (637.4)	<0.0001
uACR ^a^ (mg/g)	24.0 (14.0; 48.0)	24.0 (570.2)	24.0 (569.6)	0.9
Uric acid ^a^ (mg/dL)	5.4 (4.3; 6.6)	5.2 (555.8)	5.4 (588.2)	0.09
Diabetes complications ^b^				
Retinopathy	370 (32.5%)	216 (33.7%)	154 (30.9%)	0.321
CKD	224 (19.7%)	135 (21.1%)	89 (17.9%)	0.179
DSPN	834 (73.2%)	511 (79.7%)	323 (64.9%)	<0.0001
Coronary artery disease	479 (42.1%)	227 (35.4%)	252 (50.6%)	<0.0001
Cerebrovascular disease	105 (9.2%)	58 (9%)	47 (9.4%)	0.821
Peripheral artery disease	178 (15.6%)	72 (11.2%)	106 (21.3%)	<0.0001
Comorbidities ^b^				
Arterial hypertension	978 (85.9%)	576 (89.9%)	402 (80.7%)	<0.0001
Overweight and obesity	983 (86.3%)	574 (89.5%)	409 (82.1%)	0.0003

BMI = body mass index; HbA1c = glycated hemoglobin; LDLc = low-density lipoprotein cholesterol; HDLc = high-density lipoprotein cholesterol; TGs = triglycerides; BUN = blood urea nitrogen; eGFR = estimated glomerular filtration rate; uACR = urine albumin–creatinine ratio; CKD = chronic kidney disease; DSPN = distal symmetric polyneuropathy. ^a^ Continuous variables are indicated by their median (interquartile range) or (average rank); ^b^ categorical variables are presented by the number of individuals and absolute frequency (percentage) in the sample; * for comparison between genders.

**Table 2:** Comparison of demographic and clinical features of T2D patients based on UTI status.

Variable ^a^	Patients with UTIs (n = 225)		Patients without UTIs (n = 914)		*p*-Value *
	Median	Average Rank	Median	Average Rank	
Age (years)	70.0	715.1	62.0	534.2	<0.0001
Diabetes duration (years)	13.0	675.2	9.0	544.0	<0.0001
BMI (kg/m^2^)	31.0	616.5	30.0	557.9	0.01
HbA1c (%)	9.0	752.1	8.0	525.1	<0.0001
LDLc (mg/dL)	99.0	533.8	110.0	578.9	0.06
TGs (mg/dL)	168.0	579.1	167.5	567.7	0.6
HDLc (mg/dL)	40.0	528.3	42.0	579.5	0.03
BUN (mg/dL)	44.0	628.0	40.0	555.7	0.003
Serum creatinine (mg/dL)	1.0	618.2	0.9	558.1	0.01
Uric acid (mg/dL)	5.6	625.6	5.3	556.2	0.004
eGFR (mL/min/1.73 m^2^)	74.0	525.6	76.0	580.9	0.02
uACR (mg/g)	26.0	611.5	23.0	559.7	0.03

BMI = body mass index; HbA1c = glycated hemoglobin; LDLc = low-density lipoprotein cholesterol; HDLc = high-density lipoprotein cholesterol; TGs = triglycerides; BUN = blood urea nitrogen; eGFR = estimated glomerular filtration rate; uACR = urine albumin–creatinine ratio. ^a^ Continuous variables; * Mann–Whitney U test.

**Table 3:** Diabetes-related complications and comorbidities in patients with and without UTIs.

Variable ^b^	Patients with UTIs % (n)	Patients Without UTIs % (n)	*p*-Value *
Retinopathy	39.6% (89/225)	30.7% (281/914)	0.01
CKD	40.4% (91/225)	14.6% (133/914)	<0.0001
DSPN	94.2% (212/225)	68.1% (622/914)	<0.0001
Coronary artery disease	40.4% (91/225)	42.5% (388/914)	0.58
Cerebrovascular disease	23.1% (52/225)	5.8% (53/914)	<0.0001
Peripheral artery disease	15.1% (34/225)	15.8% (144/914)	0.1
Hypertension	92.9% (209/225)	84.1% (769/914)	0.0007
Overweight and obesity	88.8% (200/225)	85.6% (783/914)	0.2

CKD = chronic kidney disease; DSPN = distal symmetric polyneuropathy. ^b^ Categorical variables; * Chi-squared test.

**Table 4:** Risk factor analysis for UTIs in patients with T2D.

Parameter	OR	95% CI	*p*-Value
Age (years) *	1.05	1.03–1.07	<0.0001
Diabetes duration (years) *	1.04	1.02–1.06	<0.0001
BMI (kg/m^2^) *	1.05	1.02–1.07	0.0002
HbA1c (%) *	1.58	1.40–1.77	<0.0001
Female gender	3.47	2.46–4.88	<0.0001
Retinopathy	1.47	1.09–1.99	0.01
CKD	3.98	2.88–5.51	<0.0001
DSPN	7.65	4.29–13.63	<0.0001
Cerebrovascular disease	4.88	3.22–7.40	<0.0001
SGLT2 inhibitors treatment	1.28	0.91–1.80	0.1

BMI = body mass index; HbA1c = glycated hemoglobin; CKD = chronic kidney disease; DSPN = distal symmetric polyneuropathy; SGLT2 = sodium-glucose co-transporter 2; OR = odds ratio, CI = confidence interval. Significance level *p* < 0.0001; * Nagelkerke R^2^ = 0.19 for logistic regression analysis model.

## Data Availability

The original contributions presented in this study are included in the article. Further inquiries can be directed to the corresponding author(s).
